# *Borrelia miyamotoi* Disease in an Immunocompetent Patient, Western Europe

**DOI:** 10.3201/eid2409.180806

**Published:** 2018-09

**Authors:** Dieuwertje Hoornstra, Joris Koetsveld, Hein Sprong, Alexander E. Platonov, Joppe W. Hovius

**Affiliations:** Academic Medical Center, Amsterdam, the Netherlands (D. Hoornstra, J. Koetsveld, J.W. Hovius);; National Institute for Public Health and the Environment, Bilthoven, the Netherlands (H. Sprong);; Central Research Institute of Epidemiology, Moscow, Russia (A.E. Platonov)

**Keywords:** tickborne diseases, *Borrelia* *miyamotoi*, *Borrelia* infections, relapsing fever, serology, vector-borne infections, the Netherlands, Europe, bacteria

## Abstract

*Borrelia miyamotoi* disease is a hard tick–borne relapsing fever illness that occurs across the temperate climate zone. Human *B. miyamotoi* disease in immunocompetent patients has been described in Russia, North America, and Japan. We describe a case of *B. miyamotoi* disease in an immunocompetent patient in western Europe.

A 72-year-old woman in the Netherlands sought treatment in her third day of fever (<38.6°C) and reported myalgia, arthralgia, headache, and a 2.5-kg weight loss. Three weeks earlier she had noticed a tick bite after gardening. Several days later, an erythematous lesion appeared, increasing to palm size within 1.5 weeks and dissolving in a similar period. Full medical history was not suggestive of other causes of fever. Her previous medical history included cervical carcinoma and breast cancer, curatively treated.

Physical examination showed a moderately ill patient with a temperature of 36.7°C, heart rate of 59 bpm, blood pressure of 100/72 mmHg, an erythematous skin lesion (1.5 cm in diameter) on the thigh, and mild generalized lymphadenopathy. Initial laboratory tests revealed increased C-reactive protein (22.7 mg/L), leukopenia (2.1 × 10^9^ cells/L), elevated monocytes (11%), and thrombocytopenia (144 × 10^9^ platelets/L) (reference ranges in Technical Appendix Table 1). All other test results, including urinalysis, were unremarkable. Molecular tests of blood and skin biopsy and serologic testing for *Borrelia burgdorferi* sensu lato and syphilis were repeatedly negative, except for a C6 EIA IgM/IgG seroconversion (Immunetics, Boston, MA, USA) in convalescent-phase serum samples that was positive but could not be confirmed by either IgM or IgG immunoblot (Mikrogen, Neuried, Germany) (Technical Appendix Table 2). We did not admit the patient to the hospital, and we did not initiate antimicrobial drug treatment because her symptoms had largely resolved. At a 2-month follow-up visit, the patient had fully recovered, and laboratory test results were normal.

On the basis of the patient’s description, we suspect that she was bitten by an *Ixodes ricinus* tick, the most prevalent tick species in western Europe (*1*), which can potentially carry several tickborne pathogens: *Borrelia burgdorferi* s.l., *B.* *miyamotoi*, *Rickettsia helvetica* and *R. monacensis*, *Anaplasma phagocytophilum*, *Babesia divergens* and *B. microti*, *Neoehrlichia mikurencis*, and tick-borne encephalitis virus (*2*). Specific molecular and serologic diagnostic tests for all of these pathogens were negative, expect for 1 (false-positive) tick-borne encephalitis virus IgM ELISA result in convalescent-phase serum samples (Technical Appendix Table 2).

*B. miyamotoi*, a relapsing fever *Borrelia* species uniquely found in *Ixodes* spp. ticks in Eurasia and North America, is the causative agent of *Borrelia miyamotoi* disease (BMD), a tickborne febrile disease (*3*,*4*). Diagnosis of BMD relies on detection of spirochetes by quantitative PCR of blood and experimental serology based on glycerophosphodiester phosphodieasterase (GlpQ) antigen detection (*3*,*5*). GlpQ is present in relapsing fever *Borrelia* but not in *B. burgdorferi* s.l. and therefore can discriminate between the 2 types (*4*). In a well-described cohort of PCR-positive patients in Russia, characteristic clinical symptoms were fever, myalgia, nausea, and headaches; laboratory findings showed thrombocytopenia and diffuse organ damage (*3*).

In this patient, results of pan–relapsing fever *Borrelia* PCR and *B. miyamotoi–*specific PCR (*6*) of blood drawn at the day of clinical visit were negative. However, the fever and symptoms had subsided, which probably impeded these direct diagnostic tests. We tested for anti-GlpQ and anti–variable major proteins (Vmps) IgM and IgG using ELISA and Western blot in serum samples taken on the day of the hospital visit (3 days after disease onset), after 5 weeks (38 days), and after 3 months (88 days). Results demonstrated a clear seroconversion for predominantly IgG against GlpQ (Figure). We had previously shown that Vmps are highly immunogenic in patients with BMD (*7*) and that the presence of antibodies against GlpQ combined with antibodies against Vmps had 100% specificity for IgM and 98.3% for IgG (*8*). In this case, we could demonstrate antibodies against multiple Vmps over time (Figure). Finally, our findings were further confirmed by preferential IgM and IgG reactivity to lysates of the *B. miyamotoi* strain HT31 (tick isolate, Japan) and Izh-16 (clinical isolate, Russia) compared with reactivity to the *B. afzelii* strain PKo (skin isolate, Germany) and *B. hermsii* HS-1 (tick isolate, United States) control lysates (Technical Appendix Figure).

**Figure:**
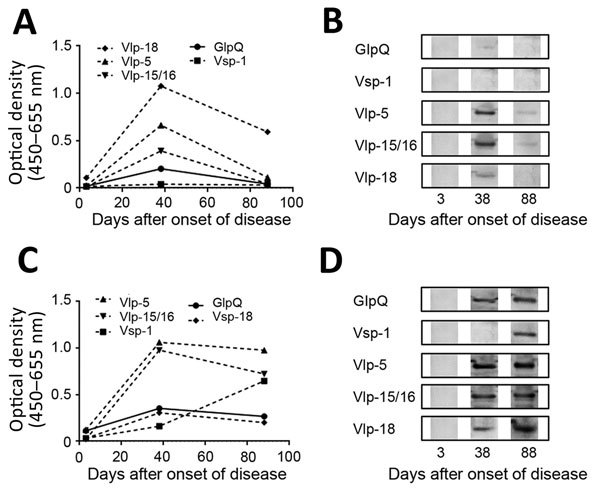
Results of GlpQ and variable major proteins (Vmps) IgM and IgG ELISA and confirmatory Western blot tests in testing of a 72-year-old woman in the Netherlands who showed evidence of *Borrelia miyamotoi* disease. A) Anti-GlpQ and anti–Vmps IgM ELISA results representative of 3 individual ELISAs. B) Confirmatory IgM Western blot results of samples taken at 3 different time points with recombinant proteins. C) Anti-GlpQ and anti–Vmps IgG ELISA results representative of 3 individual ELISAs. D) Confirmatory IgG Western blot results of samples taken at 3 different time points with recombinant proteins. GlpQ, glycerophosphodiester phosphodieasterase; Vlp, variable large protein; Vsp, variable small protein.

These findings, combined with the established presence of *B. miyamotoi* in *I. ricinus* ticks throughout Europe, clinical presentation, and laboratory findings, strongly suggest that *B. miyamotoi* was the causative agent of the patient’s symptoms. That the patient recovered even without antimicrobial treatment is consistent with a recent BMD case described in the United States (*9*). Because of the initial skin rash, we did not completely rule out *B. burgdorferi* s.l. co-infection; however, prior evaluation by an independent dermatologist, a negative *B. burgdorferi* s.l. immunoblot despite high C6 reactivity, and a negative PCR on DNA obtained from the skin biopsy argue against co-infection. Regardless, the clinical picture of fever and mild leukopenia and thrombocytopenia is compatible with BMD and not with Lyme borreliosis. Of interest, C6 reactivity in combination with a negative *B. burgdorferi* s.l. immunoblot has been described in BMD patients in the United States (*10*).

This case identifies *B. miyamotoi* as an emerging tickborne pathogen in western Europe. Because of the widespread presence of multiple other tickborne pathogens across Europe, more attention and awareness for other tickborne diseases is warranted.

Technical Appendix Additional information on diagnostic tests for 72-year-old woman in the Netherlands who showed evidence of *Borrelia miyamotoi* disease.
