# Supplementary material for: Borrelia miyamotoi Disease in an Immunocompetent Patient, Western Europe
Source: Emerg Infect Dis. 2018 Sep;24(9):1770–2. doi: 10.3201/eid2409.180806 (PMC6106421; doi:10.3201/eid2409.180806)
Supplement: Technical Appendix — Additional information on diagnostic tests for 72-year-old woman in the Netherlands who showed evidence of Borrelia miyamotoi disease. [file 18-0806-Techapp-s1.pdf]

Article DOI: https://doi.org/10.3201/eid2409.180806

Borrelia miyamotoi Disease in an Immunocompetent Patient, Western Europe

Technical Appendix

Technical Appendix Table 1. Laboratory results for patient with *Borrelia miyamotoi* disease*

Test	Result	Normal range
Chemistry		
C-reactive protein, mg/L	**22.7**	0–5
Creatinine, umol/L	78	65–95
AST, U/L	34	0–40
ALT, U/L	23	0–34
y-GT, U/L	16	0–40
Alkaline phosphatase, U/L	56	40–120
LDH, U/L	228	0–247
Total bilirubin, umol/L	6	0–17
Creatine phosphate kinase, U/L	77	0–145
Hematology		
Erythrocyte sedimentation rate, mm/h	2	0–30
Leukocyte, /L	**2.1 × 10^9**	4–10.5 × 10^9
Monocytes, %	**11**	2–9
Hemoglobin, mmol/L	8.2	7.5–10
Platelet count, /L	**144 × 10^9**	150–400 × 10^9

*Laboratory findings at initial visit. Bold indicates values outside the normal range. AST, aspartate transaminase; ALT, alanine transaminase; y-GT, gamma-glutamyl transferase; LDH, lactate dehydrogenase.

Technical Appendix Table 2. Specific testing for tickborne infectious diseases*

Test	Result	Time point, d†
Borrelia burgdorferi s.l.		
Serology (C6-EIA IgM/IgG)‡	Neg/ **Pos**/**Pos**	3 + **38** + **88**
Lyme-index	NA/**11.33**/**4.14**	3 + **38** + **88**
Western blot IgM and IgG§	Neg/Neg/Neg	3 + 38 + 88
Rickettsia spp.		
Serology (IFA IgM/IgG)	Neg/Neg	3 + 38
R. conorii	Neg/Neg	3 + 38
R. typhi	Neg/Neg	3 + 38
PCR	Neg	3
Neoehrlichia mikurensis		
PCR	Neg	3
Anaplasma phagocytophilum		
Serology (IFA IgM/IgG)	Neg/Neg	3 + 38
PCR	Neg	3
Tick-borne encephalitis virus		
Serology (IgM)	Neg/Weak **Pos**/Weak **Pos**¶	3 + **38** + **88**
(IgG)	Neg/Neg/Neg	3 + 38 + 88
PCR	Neg	3
Babesia spp.		
Serology (IFA IgM/IgG)#		
B. microti	Neg/Neg	3 + 38
PCR *B. microti*	Neg	3

*Results of conventional diagnostic tests for tick-borne infectious diseases. Bold indicates positive result. Serologic diagnostic tests for other tickborne pathogens routinely used for clinical purpose in the Netherlands were performed at either the Dutch National Institute for Public Health and the Environment (RIVM) or Erasmus Medical Center in Rotterdam. EIA, enzyme immunoassay; IFA, immunofluorescence assay.
†No. of days after onset of disease.
‡Immunetics (www.immunetics.com)
§Mikrogen (www.mikrogen.de)
¶This was interpreted as a false-positive result because of the lack of dynamics and specific IgG antibodies.
#Fluorescence microscopy.

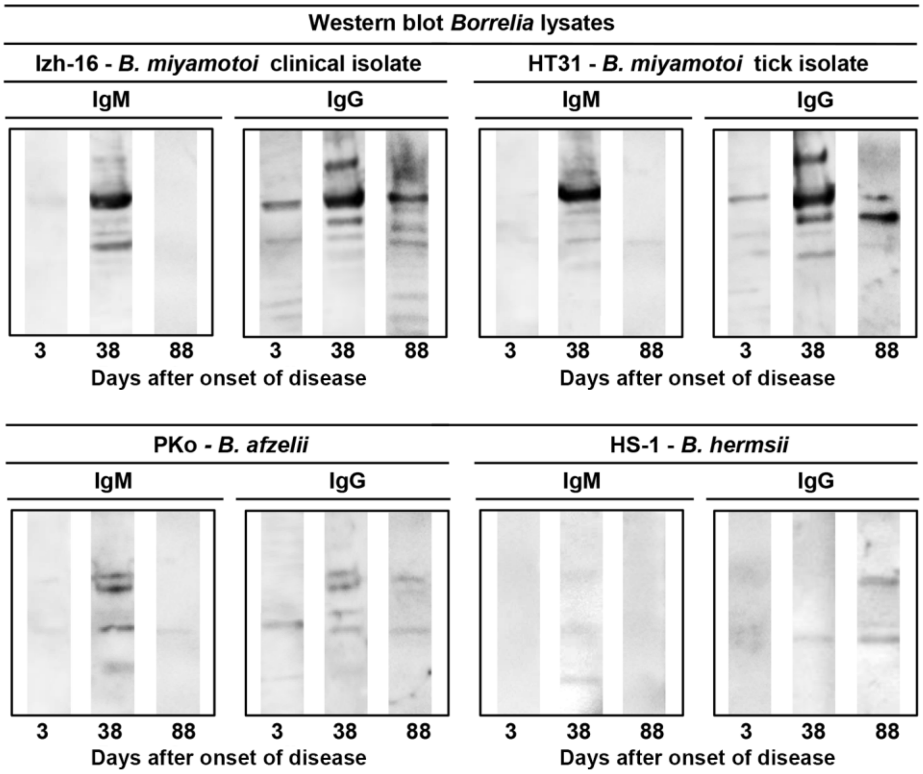

Technical Appendix Figure. Western blot results of IgM and IgG reactivity for *Borrelia* lysates. Depicted is reactivity against the clinical isolate of Izh-16 strain of *Borrelia miyamotoi*, tick isolate of HT31 strain of *B. miyamotoi*, skin isolate of PKo strain of *B. afzelii,* and tick isolate of HS-1 strain of *B. hermsii.*
